# Initial Experience of Noninvasive Quantification of Pulmonary Congestion Utilizing the Remote Dielectric Sensing System in Pediatric Patients with Heart Failure

**DOI:** 10.3390/jcm14041292

**Published:** 2025-02-15

**Authors:** Mako Okabe, Teruhiko Imamura, Mami Nishiyama, Hideyuki Nakaoka, Keijiro Ibuki, Sayaka Ozawa, Keiichi Hirono

**Affiliations:** 1 Department of Pediatrics, University of Toyama, Toyama 930-0194, Japan; makoo@med.u-toyama.ac.jp (M.O.);; 2 Second Department of Internal Medicine, University of Toyama, Toyama 930-0194, Japan; te.imamu@gmail.com

**Keywords:** cardiovascular disease, pulmonary edema, heart failure, monitoring

## Abstract

**Background/Objectives**: Remote dielectric sensing (ReDS) is a recently developed, noninvasive, electromagnetic energy-based technology designed to quantify pulmonary congestion without requiring expert techniques in adult patients with heart failure. However, its applicability in pediatric patients remains unknown. **Methods**: ReDS values and chest X-rays were simultaneously obtained from pediatric patients with a history of Fontan surgery at an outpatient clinic. The Congestion Severity Index (CSI) was calculated from chest X-rays to analyze its correlation with ReDS values. **Results**: A total of 21 pediatric patients (median age: 17 years; median height: 152.7 cm; median weight: 48.6 kg; 12 male patients) were included. ReDS values were successfully measured in all participants without any measurement failure. A mild correlation was observed between ReDS values and CSIs (r = 0.47, *p* = 0.030). In patients with ReDS values exceeding 35% (N = 11), a stronger correlation was noted between ReDS values and CSIs (r = 0.61, *p* = 0.046). In patients with ReDS values ≤ 35% (N = 10), ReDS values exhibited a wide distribution (25% to 35%) despite low CSI values. **Conclusions**: The ReDS system demonstrates potential as a feasible technology for the noninvasive quantification of pulmonary congestion in pediatric patients, irrespective of the severity of congestion. Notably, the ReDS system may have the potential to identify subclinical pulmonary congestion in pediatric patients with heart failure.

## 1. Introduction

Chest X-ray (CXR) is among the most practical modalities for evaluating the severity of pulmonary congestion in routine clinical practice [1]. Severe pulmonary congestion is often straightforward to diagnose, as it typically presents with overt symptoms such as dyspnea. In these cases, CXR frequently reveals hallmark signs of pulmonary congestion, such as the butterfly shadow [2]. Conversely, diagnosing mild pulmonary congestion using CXR alone is more challenging and necessitates expert interpretation [3].

**Figure 1:**
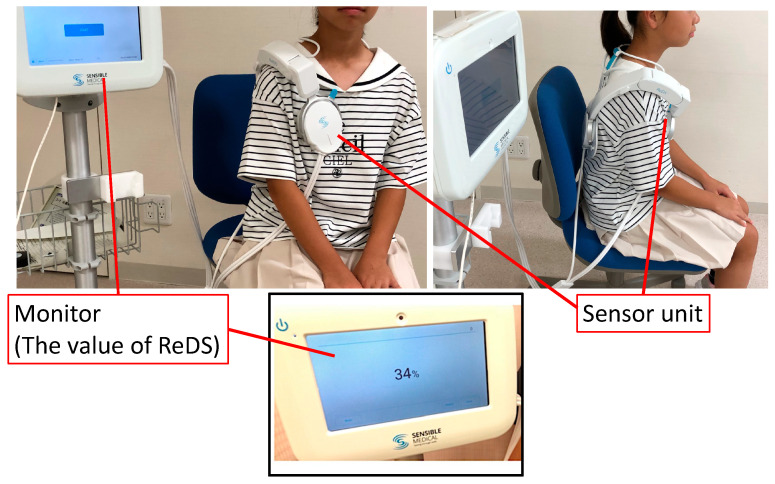
The actual instruction of the ReDS value measurement. ReDS, remote dielectric sensing. The device consists of a monitor, which is necessary for data input and the presentation of results, and a sensor unit. Patients are asked to sit on chairs and wear sensor devices. The instructors input the patients’ data, such as age, body height, and body weight. Following an approximate one-minute measurement, the ReDS value, a representative value of the amount of lung fluid amount as a percentage of the whole lung, is displayed on the monitor screen. During the measurement, patients are asked to breathe naturally without any breath holding. Measurement is possible over thin clothing, so patients do not need to take off all their clothing.

Remote dielectric sensing (ReDS) has been developed as a noninvasive technique to quantify pulmonary congestion without requiring specialized expertise (Figure 1) [4]. Its efficacy has been validated through comparisons with other modalities, including high-resolution computed tomography and invasive hemodynamic assessments [5]. Given its high sensitivity, ReDS may be particularly advantageous for detecting or excluding mild pulmonary congestion, which is a condition that is often difficult to diagnose accurately. Recent studies have demonstrated the superiority of ReDS technology over conventional CXR in adult patients with mild pulmonary congestion [6]. For instance, while it is frequently challenging to detect subclinical pulmonary congestion via CXR alone, ReDS provides quantitative measurements of lung fluid regardless of the severity of pulmonary congestion. Conversely, in cases of severe pulmonary congestion, both modalities have shown a strong correlation.

Considering these factors, ReDS technology may represent an ideal modality for assessing pulmonary congestion in pediatric patients. It is noninvasive, requires no specialized technical expertise, and has the capability to quantify lung fluid volume [7]. These features are particularly advantageous for the pediatric population, as invasive modalities are often impractical, and the accurate assessment of pulmonary congestion using conventional techniques is more challenging in children than in adults [8]. Feasible and noninvasive modalities to assess pulmonary congestion are particularly crucial to managing pediatric patients with heart failure, who have unique and specific therapeutic/management strategies.

Nevertheless, to our knowledge, no studies have evaluated the feasibility of ReDS measurements in pediatric cohorts. Notably, the ReDS system was originally designed for adult patients, and its applicability to pediatric patients, who have smaller physiques and may be too small to use the ReDS device effectively, remains uncertain [4].

In this prospective proof-of-concept study, we aimed to (1) evaluate, for the first time, the feasibility of ReDS measurements in pediatric patients and (2) compare the diagnostic performance of CXR and ReDS in assessing pulmonary congestion in pediatric patients with a history of Fontan surgery, which is a condition that remains a significant unresolved cause of heart failure in this population.

## 2. Materials and Methods

### 2.1. Patient Selection

Pediatric patients with a history of Fontan surgery who received outpatient follow-up in a clinically stable condition between 2023 and 2024 were deemed eligible for inclusion in the present study. Patients weighing over 30 kg and those who consented to undergo ReDS measurements were included. Exclusion criteria comprised patients unable to wear the ReDS system, those who declined to participate in the study, those with active pulmonary diseases such as lung cancer or pneumonia, and those with a history of lung surgeries. All patients underwent ReDS value measurement as described below. CXR was obtained simultaneously on the same day.

All participants provided written informed consent prior to their enrollment in the study. The study protocol conformed to the ethical principles delineated in the Declaration of Helsinki and received approval from the Ethics Committee of the University of Toyama (MTK2020007).

### 2.2. Measurement of ReDS

The ReDS system is a noninvasive technology developed to assess lung fluid content by analyzing the dielectric properties of lung tissue through low-power electromagnetic signals emitted via sonar [7]. ReDS measurements were acquired by positioning the patient in a resting, seated posture while wearing a specialized vest for a duration of one minute (Figure 1). According to the manufacturer’s specifications, the normal ReDS value range is between 20% and 35%; however, these parameters have not been extensively validated in real-world clinical contexts, particularly in pediatric populations. Generally, the reproducibility of ReDS is good. The manufacturer of the ReDS system reports that the instrument’s error is approximately 2–3% regarding variability in the measurements. In this study, the same examiner performed the measurements following the same procedure. All subjects were measured in a seated position while wearing light clothing.

### 2.3. Measurement of Congestion Score Index from Chest X-Ray

**Figure 2:**
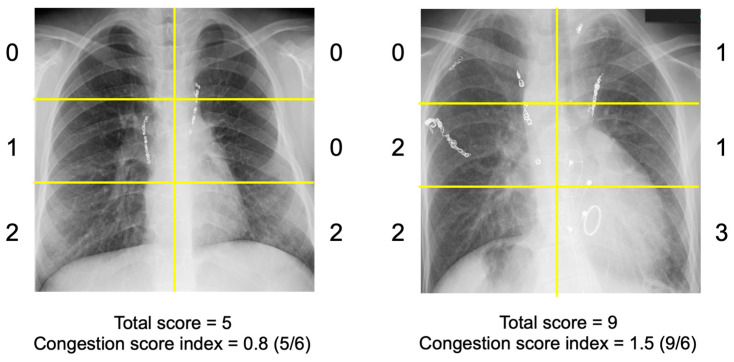
How to measure the CSI of a chest X-ray. Congestion scoring was conducted for each of the six lung field segments to provide a comprehensive assessment of pulmonary congestion. The grades of congestion were defined as follows: 0 = normal, 1 = cephalization, perihilar haze, peribronchial cuffing, or Kerley lines; 2 = interstitial pulmonary edema and localized or confluent mild edema; 3 = confluent intense edema. The total congestion score was calculated by summing the individual scores for all six lung segments. In cases where a lung segment was obscured or completely covered by pleural effusion, that segment was excluded from the scoring process to ensure the accurate representation of lung field conditions. To standardize the assessment across individuals with varying numbers of evaluable segments, the congestion score index was derived. This index was calculated by dividing the total congestion score by the number of lung segments that were available for evaluation, providing a normalized metric for the extent of pulmonary congestion.

The severity of radiographic pulmonary congestion on CXR was assessed using the previously proposed Congestion Severity Index (CSI) (Figure 2) [9,10]. Congestion was assessed for each of the six lung field segments, with congestion grades defined as follows: 0 = normal; 1 = cephalization, peribronchial cuffing, perihilar haze, or the presence of Kerley lines; 2 = interstitial pulmonary edema, or localized/confluent mild edema; 3 = confluent severe edema.

A total score ranging from 0 to 18 was calculated. If a segment was obscured by pleural effusion, it was excluded from scoring. The CSI was determined by dividing the total congestion score by the number of scorable lung segments. Two independent, experienced physicians (S.O. and H.N.), blinded to the clinical parameters, including ReDS values, evaluated the CSI for all patients, and the final CSI values were obtained by averaging their assessments to minimize inter-observer variability. The cardio-thoracic ratio (CTR) was also measured in all patients using the obtained CXR following the standard process.

### 2.4. Statistical Analysis

Continuous variables were expressed as medians and interquartile ranges due to the small sample size. Categorical variables were presented as frequencies and percentages. Pearson’s correlation coefficient was employed to evaluate the relationships between ReDS values and CSI. In a subgroup analysis, the correlations between these variables were reassessed in cohorts with ReDS values exceeding 35% and those with ReDS values ≤ 35%. The correlation between ReDS values and CTR was also analyzed in the same manner. Statistical analyses were conducted using JMP Pro 17, and two-sided *p*-values < 0.05 were considered statistically significant.

## 3. Results

### 3.1. Baseline Characteristics

A total of 21 patients were included in the study (Table 1). The cohort comprised nine patients with single ventricle, five patients with tricuspid atresia, four patients with hypoplastic left heart syndrome, one patient with double outlet right ventricle, one patient with mitral atresia, and one patient with pulmonary atresia with an intact ventricular septum.

The median age of the patients was 17 years, with a median body height of 153 cm and a median body weight of 48.6 kg. ReDS measurements were successfully obtained in all participants without any failures. Notably, no patients reported discomfort during the ReDS measurement process.

### 3.2. ReDS Values and CSI

**Figure 3:**
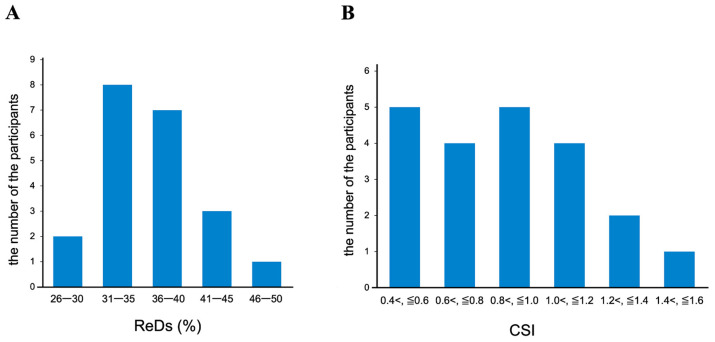
Distribution of ReDS values and CSIs. It shows the distribution of the number of people with ReDS values (**A**), It shows the distribution of the number of people with CSI (**B**). ReDS, remote dielectric sensing; CSI, congestion score index.

**Figure 4:**
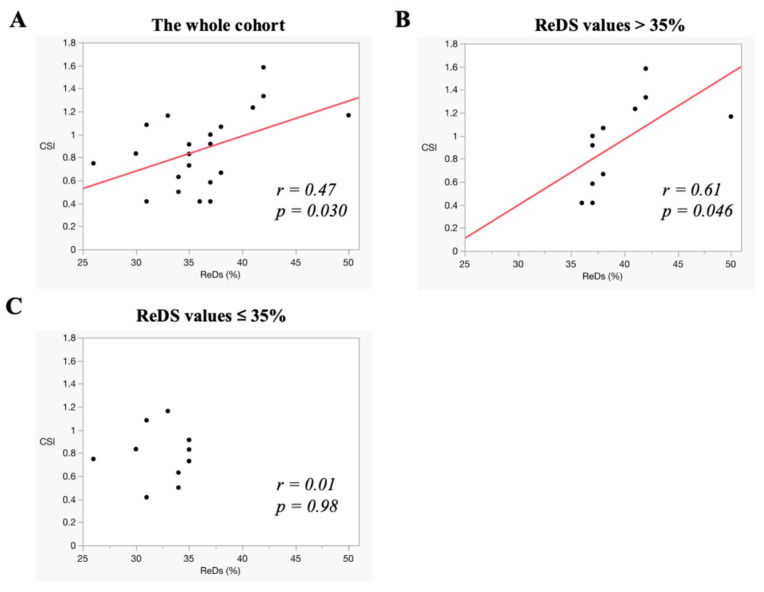
The correlation between ReDS values and CSIs. The correlations were assessed in the whole cohort (**A**), in those with ReDS values above 35% (**B**), and in those with ReDS values ≤ 35% (**C**). ReDS, remote dielectric sensing; CSI, congestion score index. The correlation between the ReDS value and CSI showed moderate scores in the whole cohort and in those with ReDS values above 35%. In the cohort with ReDS values ≤ 35%, ReDS values were distributed widely despite a relatively low CSI. The red lines represent the regression lines.

ReDS values and CSI showed a wide distribution across the participants (Figure 3A,B). A moderate correlation was observed between the ReDS values and CSI in the overall cohort (r = 0.47, *p* = 0.030) (Figure 4A). For the sub-analysis, participants were stratified into two groups: those with ReDS values exceeding 35% (N = 11) and those with ReDS values ≤ 35% (N = 10). Among participants with ReDS values above 35%, a stronger correlation between ReDS values and CSI was noted (r = 0.61, *p* = 0.046) (Figure 4B). In contrast, no significant correlation was observed between ReDS values and CSI among the participants with ReDS values ≤ 35% (r = 0.01, *p* = 0.98) (Figure 4C). ReDS values were distributed widely between 25% and 35% despite a relatively lower CSI (all CSIs were below 1.2).

### 3.3. ReDS Values and CTR

**Figure 5:**
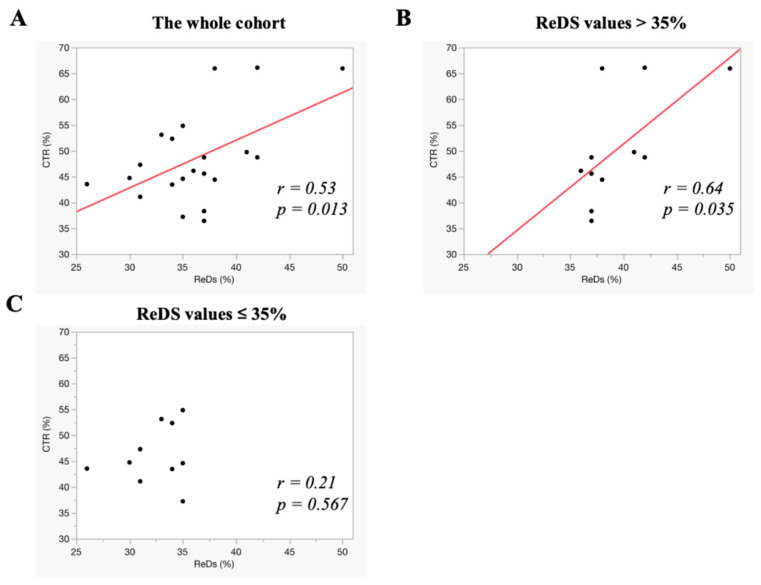
The correlation between ReDS values and CTR. The correlations were assessed in the whole cohort (**A**), in those with ReDS values above 35% (**B**), and in those with ReDS values ≤ 35% (**C**). ReDS, remote dielectric sensing; CTR, cardio-thoracic ratio. The correlation between the ReDS value and CSI was moderate in the whole cohort. However, this significant correlation disappeared in the cohort with ReDS values ≤ 35%. The red lines represent the regression lines.

ReDS values and CTR demonstrated a similar pattern of correlation. In the overall cohort, ReDS values exhibited a moderate correlation with CTR (r = 0.53, *p* = 0.013) (Figure 5A). Among the participants with ReDS values exceeding 35%, the correlation between ReDS values and CTR was stronger than in the overall cohort (r = 0.64, *p* = 0.035) (Figure 5B). Conversely, among the participants with ReDS values ≤ 35%, no significant correlation was observed between ReDS values and CTR (r = 0.21, *p* = 0.567) (Figure 5C).

## 4. Discussion

In this prospective study, we evaluated the feasibility of the ReDS system and its ability to measure ReDS values and quantify pulmonary congestion by comparing these results with CXR assessments in pediatric patients with a history of Fontan surgery. To the best of our knowledge, this proof-of-concept study represents the first clinical application of the ReDS system in a pediatric population.

The ReDS system was demonstrated to be feasible for use in pediatric patients, many of whom had a body height below the manufacturer’s recommended minimum threshold of 155 cm [4]. Importantly, no patients reported discomfort during the measurements. ReDS values were successfully obtained from all participants and showed a moderate correlation with CSIs and CTRs, respectively, which are semi-quantitative indicators of pulmonary congestion. Notably, a strong correlation was observed between ReDS values and CSIs/CTRs in patients with ReDS values exceeding 35%, which is indicative of severe pulmonary congestion. Conversely, in patients with ReDS values ≤ 35%, the ReDS system effectively stratifies the severity of pulmonary congestion, even when CSIs and CTRs are within relatively lower ranges. This highlights the ReDS system’s ability to distinguish subclinical mild congestion from the absence of congestion, which is a capability that CXR parameters cannot reliably achieve.

### 4.1. Feasibility of ReDS System in Pediatric Patients

The ReDS system was originally developed for use exclusively in adult patients [4]. To the best of our knowledge, this study is the first to evaluate the feasibility of ReDS measurements in a pediatric population. This finding is particularly significant, as pulmonary congestion is a condition observed in both adult and pediatric patients. Notably, assessing pulmonary congestion in pediatric patients is inherently more challenging than in adults [8]. Routinely and repeatedly utilizing multimodal approaches, such as CXR, echocardiography, and right heart catheterization, is often impractical in this cohort due to radiation exposure and the requirement for prolonged patient cooperation [11].

For ReDS measurements, patients are required to wear a specially designed vest optimized for adult physiology. The ReDS system necessitates approximately one minute of rest in a seated position, during which the patient must remain still. These measurements can be conducted during natural breathing without any additional effort from the patient.

Given the significant differences in body composition between pediatric and adult patients, this preliminary proof-of-concept study was designed to evaluate the feasibility of ReDS measurements in this unique population. Our findings confirm that ReDS measurements are feasible in pediatric patients when performed in accordance with institutional criteria. However, further experimental studies are needed to validate the accuracy of ReDS measurements and the usefulness of ReDS values as indicators of medication adjustment in this population.

To address device limitations, we included only pediatric patients with a body weight above 30 kg in this study. The applicability of the ReDS system in individuals weighing less than 30 kg remains uncertain. Moreover, as ReDS value measurements require one minute of rest, obtaining accurate measurements in pediatric patients who are unable to follow instructions may pose additional challenges and necessitate the development of specialized techniques.

### 4.2. ReDS Values and CXR Findings

CXR remains one of the most commonly employed tools for assessing pulmonary congestion in both adult and pediatric patients [9]. In this study, we calculated the CSI to semi-quantify the degree of pulmonary congestion observed on CXR and compared these values with ReDS measurements [10].

The correlation between ReDS values and CSIs was modest. Notably, CSI values remained low when pulmonary congestion was absent or mild (i.e., ReDS values ≤ 35%). However, ReDS values exhibited a broader range (mostly ranging between 25% and 35%), suggesting that the ReDS system may effectively stratify mild pulmonary congestion that cannot be detected by CXR alone.

Identifying mild pulmonary congestion using CXR alone can be challenging, even for board-certified experts [3]. The use of repeated CXR or computed tomography, which are additional promising modalities for assessing pulmonary congestion, is discouraged in pediatric patients due to concerns about radiation exposure [12]. Nonetheless, serial assessments of pulmonary congestion are often necessary to optimize diuretic dosages. Notably, the optimal percentage range of the water component in this population is narrower than in adults [13]. Furthermore, the recent literature has highlighted that even slight residual pulmonary congestion is a significant risk factor for poorer clinical outcomes in patients with heart failure [14]. Given its noninvasive nature, the ReDS system may be particularly advantageous for monitoring mild pulmonary congestion or detecting subclinical pulmonary congestion in pediatric patients through repeated measurements.

For instance, in daily clinical practice, the ReDS system could be utilized at discharge to assess residual pulmonary congestion or during routine outpatient visits to adjust diuretic doses [15]. In these clinical scenarios, pulmonary congestion is generally mild, as patients with severe pulmonary congestion are typically managed in intensive care units. Although we measured only one timepoint in each patient in the present study, future concerns include the ReDS-guided management of pediatric patients with heart failure by repeatedly measuring ReDS values. The advantages of ReDS technology, such as noninvasiveness, no requirement of expert technique, and the ability to identify the presence of mild pulmonary congestion and accurately quantify the degree of lung fluid, should help us establish optimal, ReDS-guided management.

Conversely, a strong linear association was observed between ReDS values and CSI/CTR in patients with evident pulmonary congestion (i.e., ReDS values above 35%). In such cases, CXR typically reveals clear signs of severe pulmonary congestion, such as a butterfly shadow pattern [2]. Furthermore, it may be challenging to make patients sit on a chair for one minute to obtain ReDS values in such a critical clinical scenario with unstable hemodynamics. Consequently, the use of ReDS measurements may not be essential in cases where pulmonary congestion is already apparent. Instead, a multimodal approach, including CXR, computed tomography, and right heart catheterization, may be employed for more detailed etiological assessments.

It is important to acknowledge that the ReDS system cannot differentiate between various conditions that may elevate ReDS values, such as pleural effusion or pulmonary pneumonia, which can be identified through other modalities, as mentioned above [7]. In the presence of these lung diseases, ReDS values are overestimated. The device is applicable for screening de novo patients or following patients in whom specific lung diseases are already excluded.

### 4.3. Study Limitations

This study is proof-of-concept, and several concerns and limitations should be acknowledged. As a single-center study, the findings necessitate further investigation to evaluate their scientific rigor and external validity. We excluded patients with a body weight below 30 kg and less cooperative patients who could not understand clinicians’ instructions. Notably, other modalities may also be challenging when applied in such cohorts. The applicability of the ReDS system in these cohorts requires further validation studies. We compared the ReDS system and CXR findings in the pediatric cohorts. This may not be sufficient to support the clinical integration of the ReDS system in this cohort. Further validation studies are essential to compare the ability to assess pulmonary congestion between the ReDS system and other modalities.

Additionally, we excluded patients with lung diseases, recognizing that ReDS technology is not an imaging modality. Therefore, it is essential to use ReDS measurements in conjunction with other imaging techniques, such as CXR, to comprehensively assess a range of cardiopulmonary conditions. Future studies should explore the utility of ReDS in diverse patient populations and investigate its role within a multimodal diagnostic framework [16,17].

Several studies have mentioned the clinical implication of ReDS-guided management, during which clinicians adjust the dose of diuretics by referencing ReDS values [15,18,19,20]. The clinical implication of ReDS measurements and its clinical benefits in pediatric patients warrant further studies.

## 5. Conclusions

The ReDS system shows promise as a feasible technology for the noninvasive quantification of pulmonary congestion in carefully selected pediatric patients, regardless of the severity of congestion. Further studies are necessary to explore the clinical implications of ReDS-guided management in pediatric patients, such as exploring ReDS thresholds for intervention and its cost-effectiveness compared to conventional imaging modalities, with the goal of optimizing therapeutic strategies and improving long-term outcomes. Future research should also focus on validating these findings across diverse patient populations and assessing the potential integration of ReDS measurements into routine clinical practice.

## Figures and Tables

**Table 1:** Baseline characteristics.

	N = 21
Demographics	
Age, years	17 (15, 20)
Men sex	12 (57.1%)
Body height, cm	152.7 (148.5, 166.5)
Body weight, kg	48.6 (40.0, 54.1)
Body mass index, kg/m^2^	20.4 (16.4, 21.2)
Laboratory data	
Plasma NT-pro B-type natriuretic peptide, pg/mL	101 (36, 214)
Chest X-ray findings	
Congestion score index	0.83 (0.64, 1.08)
Cardio-thoracic ratio	0.46 (0.43, 0.52)
ReDS value, %	36 (34, 38)

Continuous variables were expressed as the median and interquartile, and categorical variables were expressed as numbers and percentages. ReDS, remote dielectric sensing.

## Data Availability

The authors confirm that the data supporting the findings of this study are available within the article.

## Abbreviations

The following abbreviations are used in this manuscript:

ReDS	Remote dielectric sensing
CSI	Congestion Severity Index
CTR	Cardio-thoracic ratio
CXR	Chest X-ray
